# Drivers’ Visual Characteristics when Merging onto or Exiting an Urban Expressway

**DOI:** 10.1371/journal.pone.0162298

**Published:** 2016-09-22

**Authors:** Ying Cheng, Li Gao, Yanan Zhao, Feng Du

**Affiliations:** 1 School of Mechanical Engineering, Beijing Institute of Technology, Beijing, China; 2 School of Automotive & Transportation, Tianjin University of Technology & Education, Tianjin, China; University of Muenster, GERMANY

## Abstract

The aim of this study is to examine drivers’ visual and driving behavior while merging onto or exiting an urban expressway with low and high traffic densities. The analysis was conducted according to three periods (approaching, merging or exiting, and accelerating or decelerating). A total of 10 subjects (8 males and 2 females) with ages ranging from 25 to 52 years old (M = 30.0 years old) participated in the study. The research was conducted in a natural driving situation, and the drivers’ eye movements were monitored and recorded using an eye tracking system. The results show that the influence of traffic density on the glance duration and scan duration is more significant when merging than when exiting. The results also demonstrate that the number of glances and the mean glance duration are mainly related to the driving task (e.g., the merging period). Therefore, drivers’ visual search strategies mainly depend on the current driving task. With regard to driving behavior, the variation tendencies of the duration and the velocity of each period are similar. These results support building an automated driving assistant system that can automatically identify gaps and accelerate or decelerate the car accordingly or provide suggestions to the driver to do so.

## Introduction

Visual information is extremely vital to keep safe driving; Most studies [1–4] agree that visual information plays a significant role in driving. Robinson et al. [4] stated that approximately 90% of driving information was captured through the eyes. Therefore, a driver’s ability to perceive important information at optimal times is critical to maintain safe driving performance, and underlying visual strategies is primarily reflected by the drivers’ eye movements.

Many studies [5–6] examining intersections showed that measuring and analyzing drivers’ visual behaviors could provide a better understanding of the underlying cognitive mechanisms. The research results of Ball [7] showed that compared to incorrect decisions and evaluations based on properly seen information, information deficiency was the main reason for driving errors that can lead to accidents. Romoser [8] discussed the differences in scanning behavior between older and younger drivers at an intersection; the results confirmed that some difficulties older adults had in scanning intersections were due to a specific attention deficit that caused older drivers to fail to properly scan hazardous areas outside the intended path of travel. A recent in-depth study [9–10] examined environmental characteristics (traffic density and the number of important areas) had a significant effect on the drivers’ gaze and driving behavior when entering intersections. The results of that study showed that drivers’ attention allocation (e.g., mean gaze duration) and driving behavior (e.g., waiting time) depended on the environmental characteristics that required different driver actions and change in their relevance when entering an intersection.

Moreover, many studies [11–12] have been performed on curved road sections. One study described drivers’ eye movement and vehicle control behavior on a curved section of an urban motorway; the results showed that the factors that contributed to human errors were related to the motorway environment, eye movement, and centrifugal acceleration in the curved section. Other studies [13–18] assumed that drivers focused attention mainly on the occlusion point when approaching open curves on rural roads and that the working memory load lead to a significant decrease in visual anticipation in real conditions. However, recent studies [19–22] mainly focus on scanning behavior in intersections and in curved sections; little is known about drivers’ visual scanning when merging onto or exiting an urban expressway. Furthermore, these experiments are mostly carried out using a driving simulator study; whether these results can represent drivers’ visual scanning behavior and driving behavior in natural driving environments has been subject to debate.

Therefore, in this paper, we measure drivers’ glance movements and driving behavior while merging onto or exiting an urban expressway with low and high traffic density. Moreover, we conduct a experiment using high-end eye tracking devices in natural driving environments to explore the corresponding relations between known environment characteristics and visual search strategies of the merging or exiting processes. These results could provide a reference for the development of merging or exiting assist systems with respect to the recognition of merging or exiting intent and driving decisions.

## Method

### Participants

An experiment in a natural driving situation was conducted on an urban expressway in Tianjin, China. The participants were recruited through personal contacts and university mailing lists. Each participant in this manuscript has given written informed consent to publish these case details. The participants signed a consent document and were informed of the aim of experiment, which was to collect vehicle running parameters and the driver’s eye movements in a natural driving environment. The data were anonymized prior to analysis. The academic committee of Beijing Institute of Technology (an equivalent of an Institutional Review Board committee) approved the design and informed consent.

Ten subjects took part in the experiment, with 8 males and 2 females aged 25–52 years old (M 30 years old and SD 8 years). All participants had at least 5 years of driving experience and drove at least 10,000 miles per year. The subjects had no medical conditions that might affect eye movements and had normal or fully corrected eyesight. Relatively experienced subjects were chosen because experienced drivers can easily prioritize the directions to scan. This gave us the best chance of observing accurate visual strategies.

### Materials and apparatus

**Fig 1:**
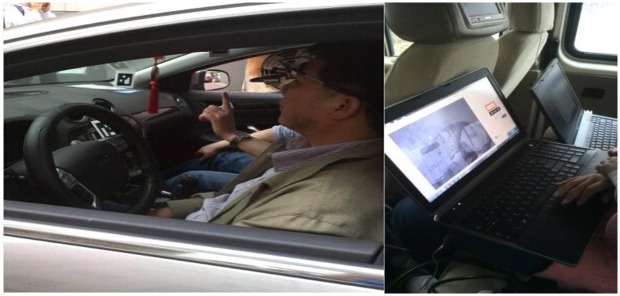
Experimental preparations and calibrating the eye tracking system. Calibration site at the university campus was used to check the accuracy of the eye tracker in between series of experiments. Beginning of experiment was selected to start near the calibration point.

The instrumented car, a Ford Mondeo 2.0 sedan with automatic transmission, was prepared for driving in real traffic conditions. The passenger seat was equipped with two notebook computers that allowed the experimenter to monitor vehicle speed, the operation of the eye-tracker and the data-logging systems. The eye movements were recorded by using a Dikablis eye tracking system that consisted of two cameras, one recording the user's eyes and the other recording the field of view. The system sampled eye position at a frequency of 50 Hz after performing calibration and had offline pupil detection functions. The travel speed and driver’s position of each subject were monitored using the vehicle traveling data recorder at all times. All data were synchronized in time using D-lab2.5 software and saved onto one notebook computer for analysis (see Fig 1).

### Procedure

The experiment route was a 11.5 km section of the Tianjin urban expressway that consisted of the two-way 6- or 8-lane main road and a two-way 4-lane auxiliary road with 60 m of total road width and a 5 m median strip. The location was 5 km away from the university campus, and the participants drove the car to the site to become familiar with the vehicle at beginning. The eye cameras were calibrated on arrival for each participant throughout the experiment.

**Fig 2:**
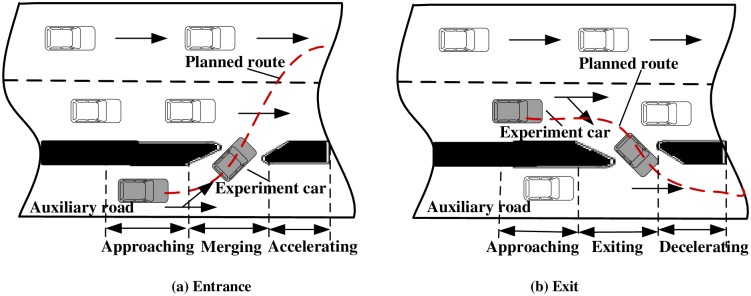
Entering or exiting an urban expressway and the three analysis periods. Red line traces the route that the subjects drove. The gray car is driven by the subject according to the planned route. White cars are traveling around the experiment car.

All driving was conducted according to the driving route in daylight. The route was divided into two recording segments: a 1.5 km length at the entrance and a 1.5 km length at the exit of the urban expressway (the entrance was on the left side of the driver, and the exit was on the right side, as shown in Fig 2). Thus, there were two recording segments per run amounting to 20 times of merging or exiting processes.

All subjects were told to drive and to respond to traffic at their own pace. A member of the research team acted as an experimenter. He seating in the back passenger seat was responsible for giving route directions, ensuring safety, monitoring the recording and performing the calibrations.

### Dependent variables

In the study, valid measurements were selected for detailed analysis. The gaze and driving data were analyzed in three periods: (1) *Approaching*, in which the driver moved straight forward to approach the entrance or exit, (2) *Merging or exiting*, in which the driver crossed through the entrance or exit, and (3) *Accelerating or decelerating*, in which the driver had completely entered the target lane (see Fig 2).

The *approaching* period was defined to begin 0.15 km before the first tangency point of the entrance or exit. The second period, *merging or exiting*, was defined to begin with the first tangency point and end with the second tangency point of the entrance or exit, over 0.10 km. Then, the *accelerating or decelerating* period was defined to begin 0.10 km in the target lane after the end of the *merging or exiting* period. Table 1 gives an overview of the variables analyzed in each period.

**Table 1:** An overview of the dependent variables analyzed in each period.

Period	Dependent variables	
	Glance data	Driving data
***Approaching***	Percentages of glanceTime and scanTime (%)	Time (s)
	Percentage of glance number (%)	Velocity (km/h)
	Mean glance duration (s)	
***Merging or exiting***	Percentages of glanceTime and scanTime (%)	Time (s)
	Percentage of glance number (%)	Velocity (km/h)
	Mean glance duration (s)	
***Accelerating or decelerating***		Velocity (km/h)

To examine how relevant environmental characteristics influence the drivers’ visual strategies and driving behavior, two environmental factors were varied: (1) the entrance located to the left, where cars were coming from the auxiliary road and conflicting with the main road traffic, and the exit located on the right, where cars were coming from the main road and driving onto the auxiliary road and (2) the event rate of the cars on the expressway with low and high traffic density. In the low traffic density situation, the average traffic density was 10.2 vehicles per km, which was equal to approximately 100 m between the cars on expressway. In the high traffic density situation, the average traffic density was approximately 45.5 vehicles per km, which was equal to a distance of approximately 22 m between cars. Therefore, a 2×2 analysis of variance (ANOVA) was conducted using traffic density and entering or exiting as within-subject factors. The date was analyzed using SPSS 19.0 and each analysis had a significance level of 0.05.

In the following, the results of the drivers’ glance and driving behavior with regard to the three periods (the approaching period, merging or exiting period, and accelerating or decelerating period) are shown. However, in the analyses of the drivers’ gaze behavior, the accelerating period was not included because the drivers’ anticipations and expectations are mainly concerned when merging onto or exiting an urban expressway, however, drivers’ gaze behavior of accelerating period cannot actively reflect the driving intention.

To examine the influence of the two environmental factors (traffic density and entrance or exit) on the drivers’ visual strategies and driving behavior when entering or exiting an urban expressway, visual scanning was classified into five driver interest regions (*DIRs*) in the driving process, with the glance angle collapsed into five categories: far left (-30° or more to the left); near left (-10° to -30°); central (-10° to +10°); near right (+10° to +30°); or far right (+30° or more to the right).

## Results

### Glance behavior

#### Percentage of glanceTime and scanTime

During the merging task, the major risk is that the vehicle could come into conflict with a vehicle in the target lane. Therefore, the vehicle on the auxiliary road will seek an acceptable gap to merge with the vehicles on the main road. Yuan et al. [23] showed that glancing, scanning and blinking are the three basic forms of eye movement. However, blinking is not involved in the visual search process, so this research bases the eye movement analyses on glance and scan behavior only.

**Fig 3:**
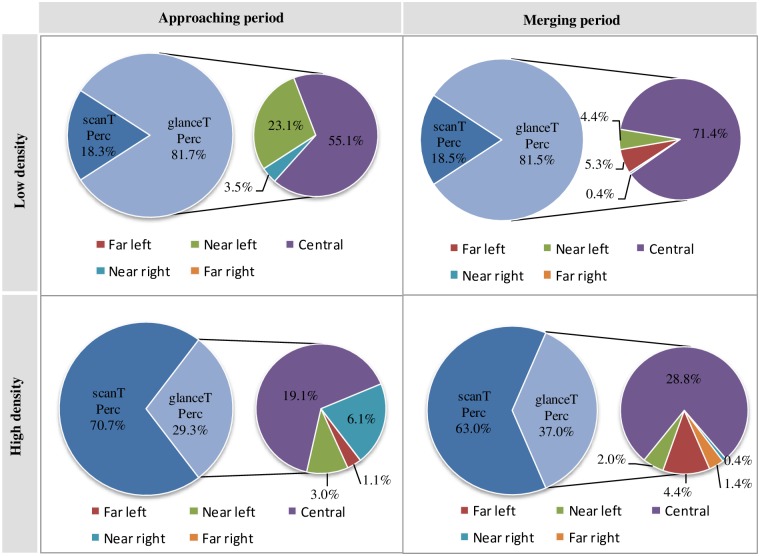
The relationship between glanceTPerc and scanTPerc at different traffic densities when entering (%). Drivers’ cognitive resources or cognitive energy consists of glanceTPerc and scanTPerc, glanceTPerc consists of 5 DIRs’ glanceTPerc during the merging task and glanceTPerc is further analysed for 5 directions (DIRs).

According to Underwood and Crundall [22], glance duration is defined as the time that the eye dwells continuously within one *DIR*, and the scan duration is the time that it takes to switch from one *DIR* to another *DIR*. Therefore, glanceTPerc is defined as the percentage of glance time during the task, which reflects the percentage of time that the subjects focus on a *DIR*. In contrast, scanTPerc is defined as the percentage of scan duration during the task, which reflects the percentage of time that the subjects are transitioning from looking at one object to another. glanceTPerc consists of one or multiple glanceTPerc of *DIRs* during the driving task, such as far left, near left, central, near right, or far right. The relationship between glanceTPerc and scanTPerc during the merging task is depicted in Fig 3.

Kahneman [24] assumed that cognitive resources or cognitive energy was limited and that humans can flexibly allocate their limited cognitive resources according to the actual need. As Fig 3 indicates, as the glanceTPerc increases during the task, the scanTPerc decreases. During merging onto the urban expressway with a high traffic density, the glanceTPerc is approximately 30%, and the scanTPerc is approximately 70%. In contrast, for the low traffic density, the glanceTPerc is approximately 80%, and the scanTPerc is approximately 20%. The results show that when the traffic density is higher, the drivers’ scanning becomes more frequent because of the larger driving recognition load; thus, scanTPerc is significantly higher (t = -6.191, p = 0.025 < 0.05) than glanceTPerc. In contrast, when the traffic density is lower, the driving recognition load is lower; thus, glanceTPerc is significantly higher (t = 4.805, p = 0.007 < 0.05) than scanTPerc with a low traffic density. Thus, the influence of traffic density on glance duration and scan duration is significant at the entrance.

In addition, based on the glanceTPerc results for each *DIR* (see small pie charts on the right of Fig 3), the drivers’ attention is mainly focused on the central and near regions (where the target lane is located) during the *approaching* period. During the *merging* period, the participants pay significantly more attention to the central and far regions, especially the rearview mirror, to avoid oncoming cars when driving from the original lane into the target lane. It can be seen that the drivers’ attention to each *DIR* was mainly influenced by the driving task.

**Fig 4:**
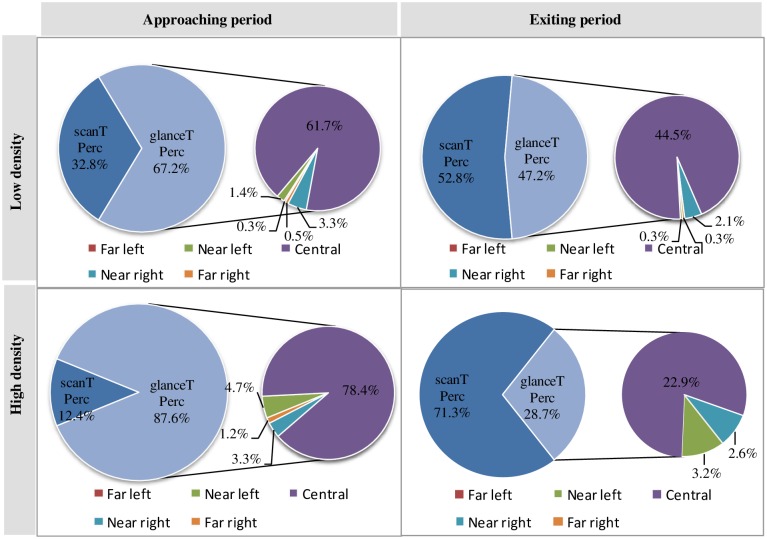
The relationship between glanceTPerc and scanTPerc at different traffic densities when exiting (%). Drivers’ cognitive resources or cognitive energy consists of glanceTPerc and scanTPerc, glanceTPerc consists of 5 DIRs’ glanceTPerc during the exiting task and glanceTPerc is further analysed for 5 directions (DIRs).

During the exiting task, the participants left the urban expressway and exited onto the auxiliary road according to the planed route. The relationship between glanceTPerc and scanTPerc during exiting is depicted in Fig 4.

As Fig 4 indicates, during the exiting task, the influence of traffic density on glanceTPerc and scanTPerc is not significant. However, glanceTPerc is higher than scanTPerc in the *approaching* period, and scanTPerc is higher than glanceTPerc in the *exiting* period regardless of traffic density. Because the speed of the vehicles on the urban expressway is generally higher than that on the auxiliary road, drivers pay more attention to vehicles in front of them before leaving the urban expressway and therefore have a higher glanceTPerc in the *approaching* period. When they leave the urban expressway and exit to the auxiliary lane, drivers increase their visual search to merge with the lower speed vehicles on the auxiliary road and therefore have a higher scanTPerc in the *exiting* period.

#### Percentage of glanceNumber

**Fig 5:**
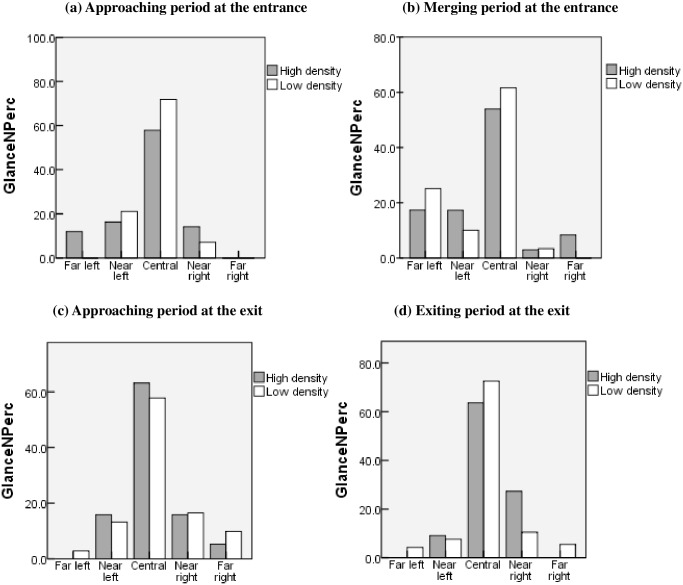
Percentage of glance numbers for each *DIR* at the entrance or exit, subdivided into the two periods.

The glanceNPerc is defined as the percentage of glance numbers at each *DIR*, which represents the probability that glances fall in each *DIR* during the task. The glanceNPerc of each *DIR* during the *approaching* period and the *merging* period at the entrance and exit is displayed in Fig 5.

Similar scanning patterns were observed among these different periods, with the majority of glance numbers occurring in the far left, near left, and central *DIR*s regardless of traffic density. As shown in the figure, the glanceNPerc shows a left-skewed distribution at each entrance period and shows a right-skewed distribution at each exit period, so the distribution of glanceNPerc is mainly based on the driving task. This indicates that the preferred region that the drivers tend to look at is the future path of the vehicle. Furthermore, the proportion of glances to the far left is found to be increasingly significant at the entrance (high density, t = 2.941, p = 0.026<0.05; low density, t = 2.568, p = 0.044<0.05), in particular, the drivers made an average of approximately 21.5% glances (SD = 5.6) to the far left (i.e., viewing the left rear view mirror) while merging into the future path of the vehicle.

However, the distribution of glanceNPerc was obviously more scattered with high traffic density, showing that drivers had a broader scan range. In contrast, with low traffic density, the distribution of glanceNPerc was relatively more concentrated, showing that drivers had a narrower scan range.

#### Mean glance duration

**Fig 6:**
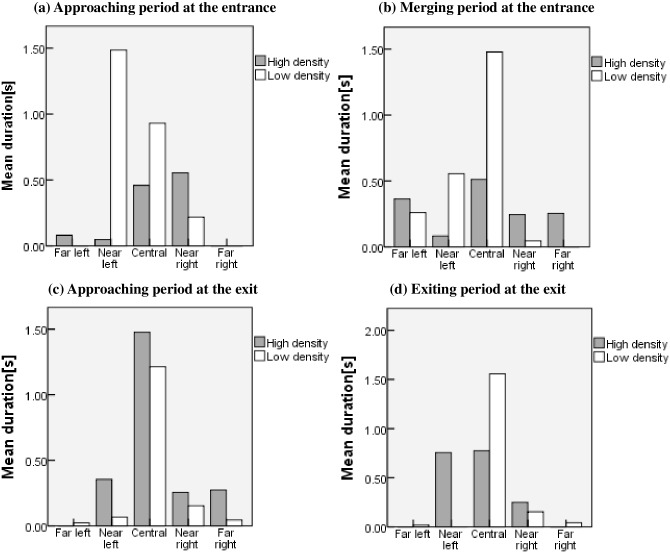
Mean glance duration of each *DIR* with high and low traffic densities at the entrance or exit.

The glanceMean is defined as the mean duration of each glance in each *DIR*, which reflects one or multiple fixation durations within each *DIR*. The glanceMean with different traffic densities at the entrance or exit is displayed in Fig 6.

Fig 6 shows a difference between the two traffic densities at the entrance; the mean duration of glances to each *DIR* with high traffic density is shorter. This indicates that when the traffic environment becomes complicated with a high traffic density, drivers have a faster search frequency to process the information; therefore, a shorter mean duration means that more cognitive effort is needed. However, when the traffic density is low, the mean durations of glances to the central and near left regions are especially longer than those of the other *DIRs*. This indicates that drivers’ glances mainly focus on the central and near left regions and that drivers less frequently scan other *DIRs*. Therefore, the longer mean duration means that less cognitive effort is needed.

Fig 6 also shows that the mean duration of glances to each DIR is similar between high and low traffic densities at the exit. There is no significant difference for the mean duration of glances to each *DIR* (high density, t = 9.941, p = 0.076>0.05; low density, t = 7.568, p = 0.054>0.05). This indicates that higher traffic volumes do not directly cause more cognitive effort when exiting an urban expressway.

### Driving behavior

#### Duration

**Fig 7:**
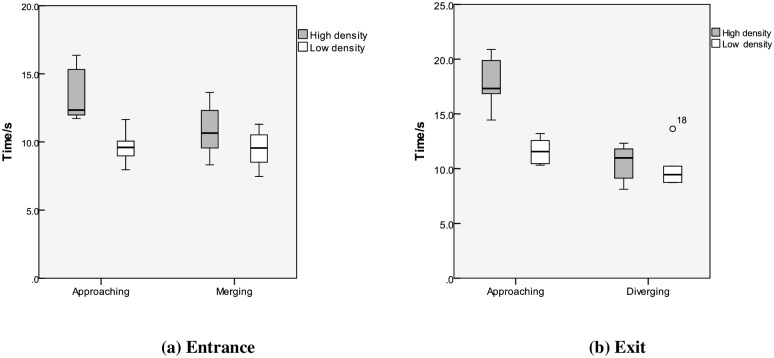
The duration of the merging or exiting process with high and low traffic densities.

As mentioned above, two steps are needed for the vehicle to complete a merging or exiting process: ① decelerate to the innermost lane, approach the merging or exiting point, and then find an acceptable merging or exiting gap; ② accelerate or decelerate smoothly to merge with vehicles in the target lane and then complete the merging or exiting process. The duration of each period in the merging or exiting process is shown in Fig 7.

As shown in Fig 7, the duration of each period in the merging or exiting process is distributed in a range. At the entrance, the *approaching* period is 9–16 s, and the *merging* period is 8–12 s. At the exit, the *approaching* period is 10–20 s, and the *exiting* period is 8–12 s. A significance test indicates that the difference in the *approaching* period duration in the high and low traffic densities is significant (F = 12.381, p = 0.013<0.05), while the differences in the *merging* period duration in different traffic densities is not significant (F = 1.108, p = 0.333>0.05). This result indicates that the driving environment with a high traffic density is more complicated, so drivers take a longer time to gather information; therefore, the influence of traffic density on the *approaching* period is significant. However, in the *merging* or *exiting* period, drivers need to complete the merging or exiting task within a certain duration limit at the entrance or exit of the urban expressway.

#### Velocity

**Fig 8:**
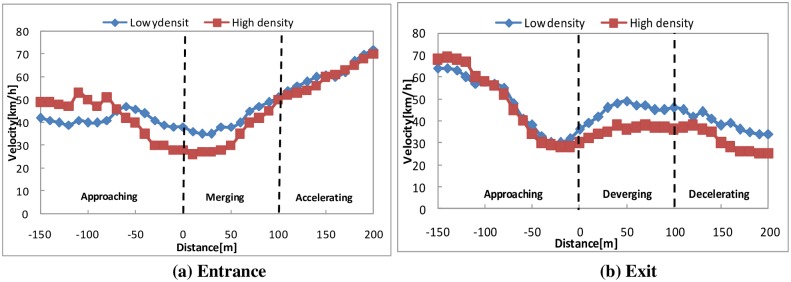
The velocity of the merging or exiting behavior with high and low traffic densities.

To analyze the driving behavior, the authors subdivided the road into three sections: approaching, merging, and accelerating for entering and approaching, exiting, and decelerating for exiting. We investigated the speed of the car while entering and exiting the urban expressway; a graph showing these three sections and the speed as a function of the distance (in meters) from the start point is shown in Fig 8.

Fig 8 shows that the variation tendency of the velocity in each period is similar. First, the participants drove at a speed of 40–50 km/h (SD = 2.6 km/h) and then began to slow down approximately 50 meters before the starting point of the entrance. Second, the participants generally drove at a speed of 30–40 km/h (SD = 3.8 km/h) when they entered the first lane of the urban expressway from the auxiliary road. Finally, the participants completed merging and accelerated to adapt to the urban expressway traffic flow.

Compared to the entrance, the participants significantly reduced their velocity from 70 km/h to 30 km/h in the first period at the exit and then generally drove at a speed of 30–40 km/h (SD = 3.8 km/h) when they left the urban expressway to the auxiliary road. After exiting, the participants decelerated to adapt to the auxiliary lane traffic flow.

In contrast, there is no significant difference (F = 7.8, p = 0.802>0.05) in the velocity between low density and high density at the entrance or exit.

A regression model was built according to the above experiment data. The speed as a function of the distance (in meters) from the start point along with indications of the three sections is shown in Table 2 for each combination of high and low traffic densities and entering or exiting tasks.

**Table 2:** Experimental regression model.

Function	Low density	High density
Entrance	$v=\{\begin{array}{ll} 40 & -150<x<0\\ 0.002x^{2}-0.075x+36.65 & 0\leq x<100\\ 37.99{\text{e}}^{0.003x} & 100\leq x<200 \end{array}$ *R*^2^ are 0.865, 0.954, and 0.952 respectively	$v=\{\begin{array}{ll} -0.002x^{2}-0.497\text{x}+20.51 & -150<x<0\\ 0.003x^{2}-0.060\text{x}+26.87 & 0\leq x<100\\ 35.28{\text{e}}^{0.003x} & 100\leq x<200 \end{array}$ *R*^2^ are 0.865, 0.740, and 0.691 respectively
Exit	$v=\{\begin{array}{ll} -0.286x\;+\;25.78 & -150<x<0\\ 0.003x^{2}-0.374\text{x}+36.25 & 0\leq x<100\\ -25.5\text{ln}(x)+159.2 & 100\leq x<200 \end{array}$ *R*^2^ are 0.865, 0.740, and 0.691 respectively	$v=\{\begin{array}{ll} -0.355x\;+\;20.4 & -150<x<0\\ 0.001x^{2}\;+\;0.221x\;+\;30.09 & 0\leq x<100\\ -19.6\text{ln}(x)+137.4 & 100\leq x<200 \end{array}$ *R*^2^ are 0.865, 0.740, and 0.691 respectively

In Table 2, *V* is the speed of merging or exiting, and *x* is the distance to the point of merging, *R*^*2*^ is correlation coefficient. The correlation coefficient *R*^*2*^ in Table 2 shows that the model is effective and can accurately reflect the relationship between speed and distance.

## Discussion

This study examines the driving strategies of car drivers in real traffic conditions when entering the first lane of a urban expressway from the auxiliary road and when exiting the urban expressway to the auxiliary road. The experimental conditions vary whether the drivers are entering or exiting the urban expressway and whether the traffic density on the urban expressway is high or low. The analysis is conducted by subdividing the road stretch into three sections: approaching, merging, and accelerating for entering and approaching, exiting, and decelerating for exiting.

In this paper, we investigate the glances time, the glances number, and mean glance duration using in-car eye trackers, and the percentage of glanceNumber reflects the switching frequency of drivers’ fixation, the percentage of glanceTime reflects the distribution of drivers’ attention, the mean glance duration reflects drivers’ cognitive load. Therefore, we believe that these three indexs can comprehensively reflect drivers’ visual behavior. The results show that as the glanceTPerc increases, the scanTPerc decreases during the task. It can be seen that drivers' gaze shifts become more frequent in high traffic density when entering the urban expressway; however, glanceTPerc and scanTPerc are not related to the traffic density when leaving the urban expressway because the vehicles are exiting and a higher traffic density cannot cause a higher cognitive load.

Furthermore, we find that while entering, subjects gaze themselves more to the left, whereas while exiting the urban expressway, they look more often to the right. This indicates that the drivers tend to look in the direction of the future path of the vehicle. The number of fixations increases with high traffic density, while the duration spends at every target decreases. This behavior is prominent in the approaching section but not very strong in the actual merging section.

When analyzing the merging or exiting driving behavior, the variation tendencies of the duration and the velocity in each period are similar. Regardless of whether entering or exiting an urban expressway, the results indicate that drivers significantly decrease their velocity in the *approaching* period, smoothly increase it in the *merging* or *exiting* period, and adjust their driving velocity according to their need in the *accelerating* or *decelerating* period. We can conclude that these findings suggest that the cognitive load is highest in the sections where the driver has to find a sufficiently long gap in the unban expressway lane to merge into a new lane and that this load decreases during the actual merging process (action phase).

Driving safely while merging onto or exiting an urban expressway depends on the drivers’ ability to find an appropriate gap and merge into the target lane. Therefore, if we can discriminate the driving intent during the process of merging onto or exiting an urban expressway, warning systems can be added at the entrance or exit to avoid collisions. Finally, the results of this study contribute to better understanding the role of drivers’ expectations and attention allocation in the causation of entering or exiting accidents. In addition, the results support building an automated driving assistant system that can automatically identify gaps and accelerates or decelerates the car accordingly or provides suggestions to the driver to do so.

## Supporting Information

S1 Fig Experimental preparations and calibrating the eye tracking system. (TIF)

S2 Fig Entering or exiting an urban expressway and the three analysis periods. (TIF)

S3 Fig The relationship between glanceTPerc and scanTPerc at different traffic densities when entering (%). (TIF)

S4 Fig The relationship between glanceTPerc and scanTPerc at different traffic densities when exiting (%). (TIF)

S5 Fig Percentage of glance numbers for each *DIR* at the entrance or exit, subdivided into the two periods. (TIF)

S6 Fig Mean glance duration of each *DIR* with high and low traffic densities at the entrance or exit. (TIF)

S7 Fig The duration of the merging or exiting process with high and low traffic densities. (TIF)

S8 Fig The velocity of the merging or exiting behavior with high and low traffic densities. (TIF)

S1 File The minimal data set showing raw eye movement data (mean glance duration of each DIR) for all participants in Experiment. (SAV)

S2 File The minimal data set showing raw diverging time date at the entrance. (SAV)

S3 File The minimal data set showing raw exiting time date at the exit. (SAV)

S1 Table An overview of the dependent variables analyzed in each period. (DOC)

S2 Table Experimental regression model. (DOC)
