# Supplementary figures and images for: Drivers’ Visual Characteristics when Merging onto or Exiting an Urban Expressway
Source: PLoS One. 2016 Sep 22;11(9):e0162298. doi: 10.1371/journal.pone.0162298 (PMC5033524; doi:10.1371/journal.pone.0162298)

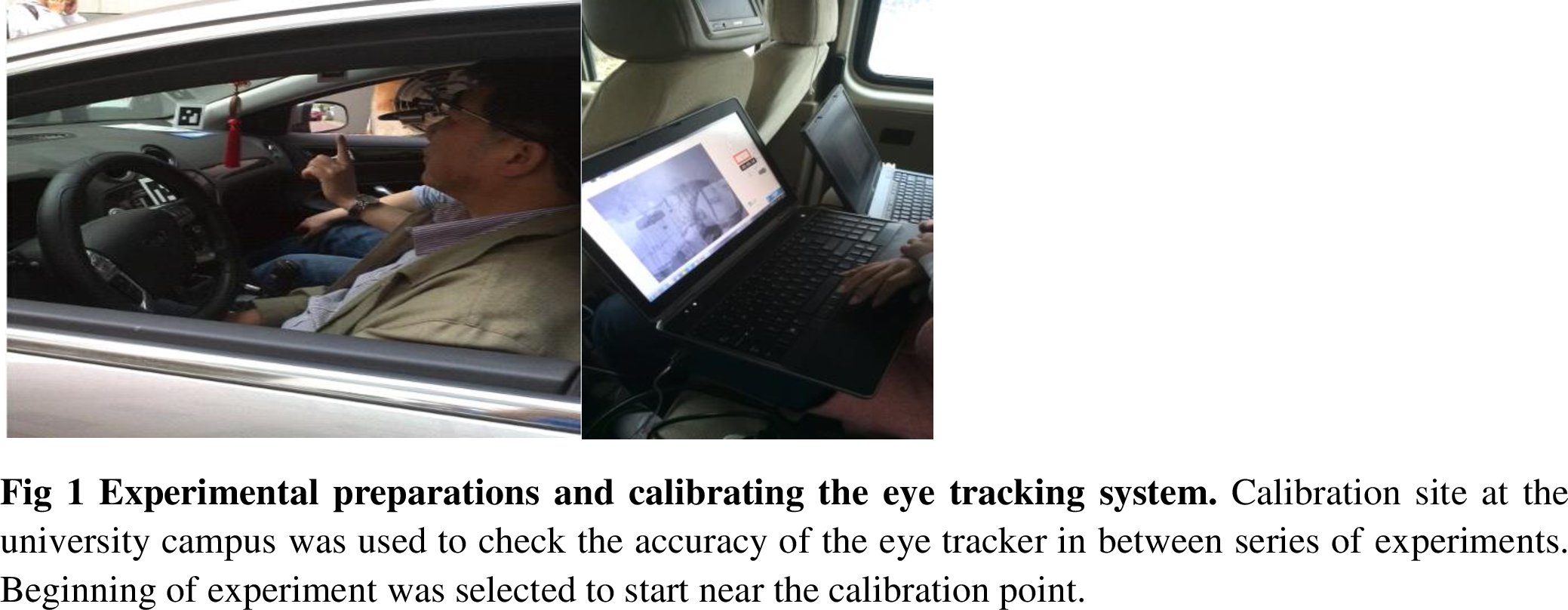

Supplement: S1 Fig — (TIF) [file pone.0162298.s001.tif]

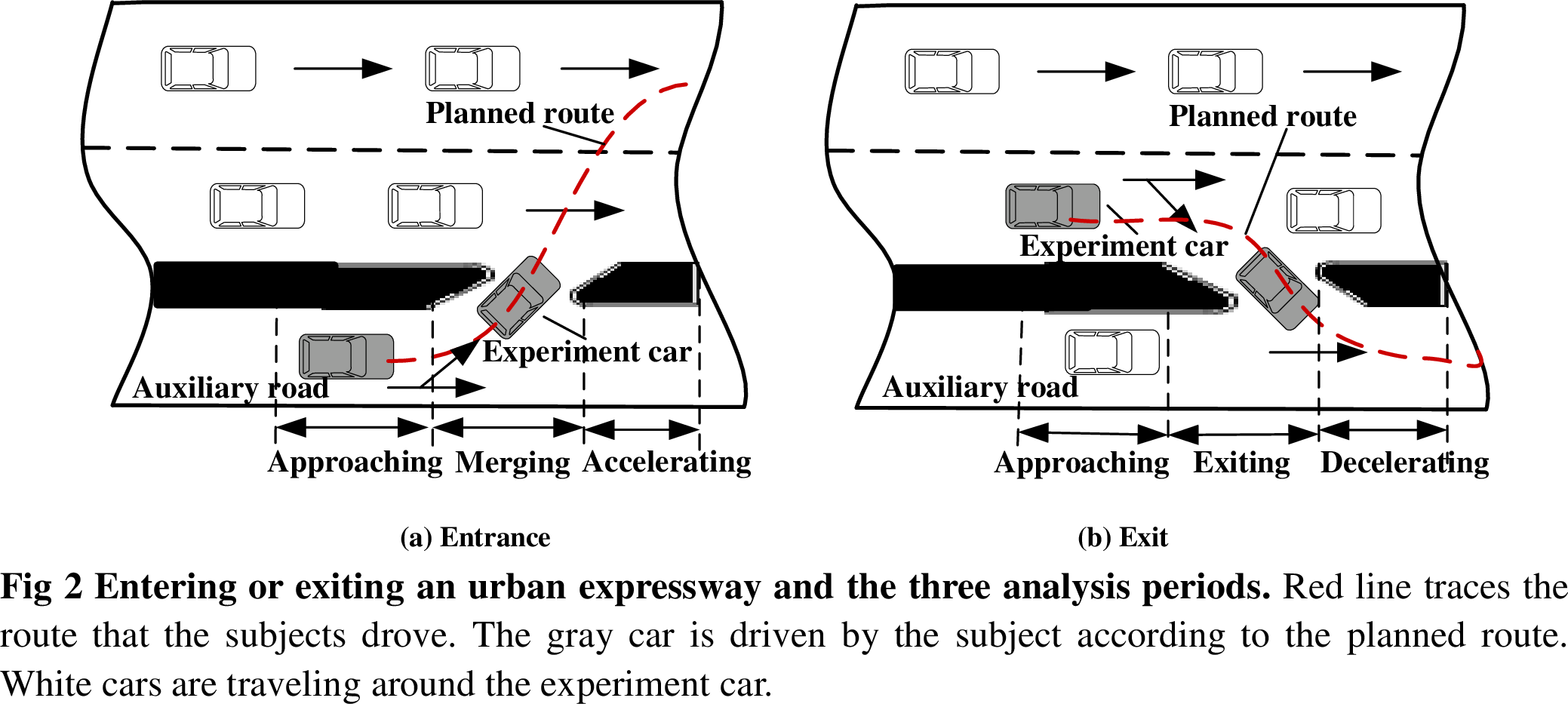

Supplement: S2 Fig — (TIF) [file pone.0162298.s002.tif]

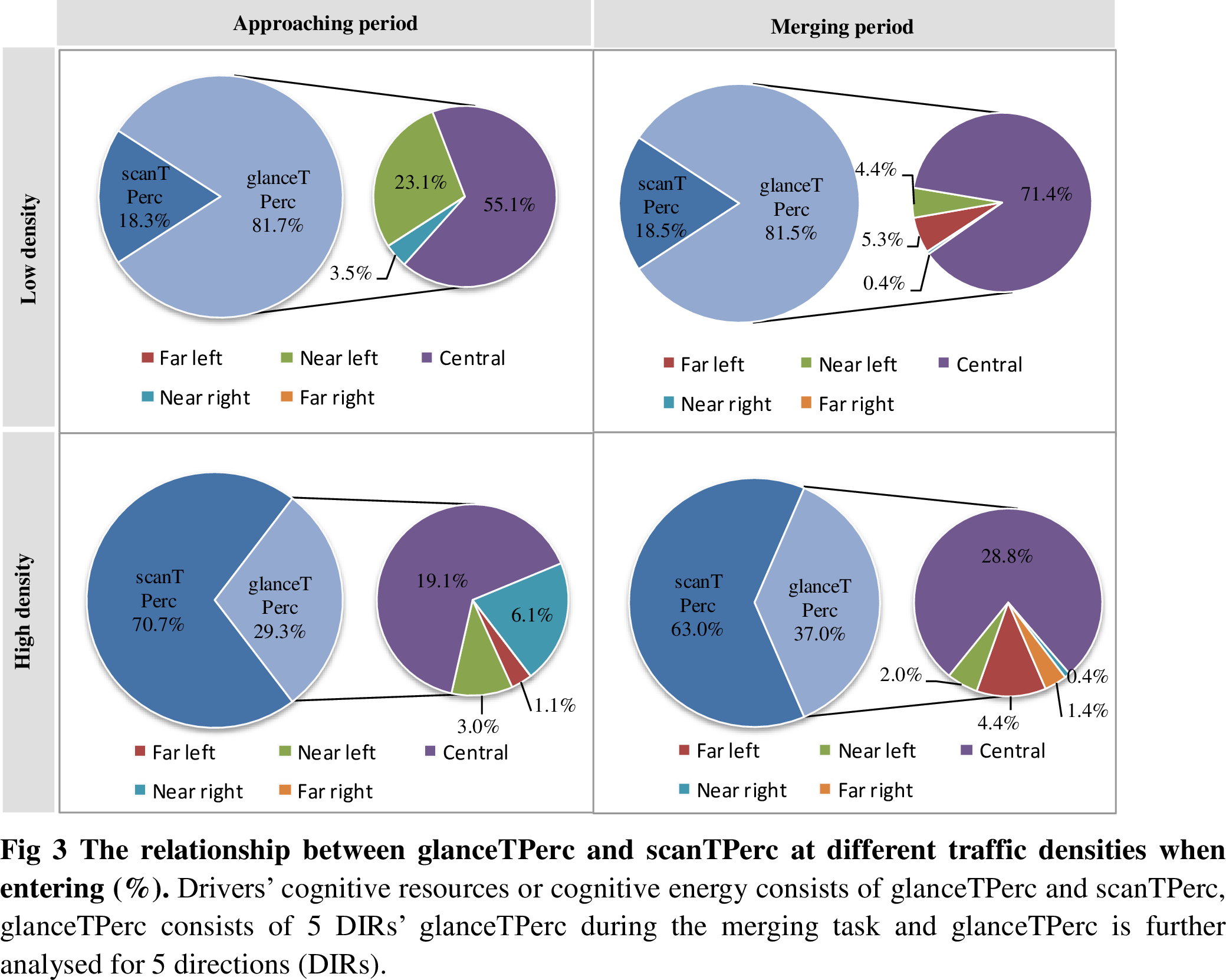

Supplement: S3 Fig — (TIF) [file pone.0162298.s003.tif]

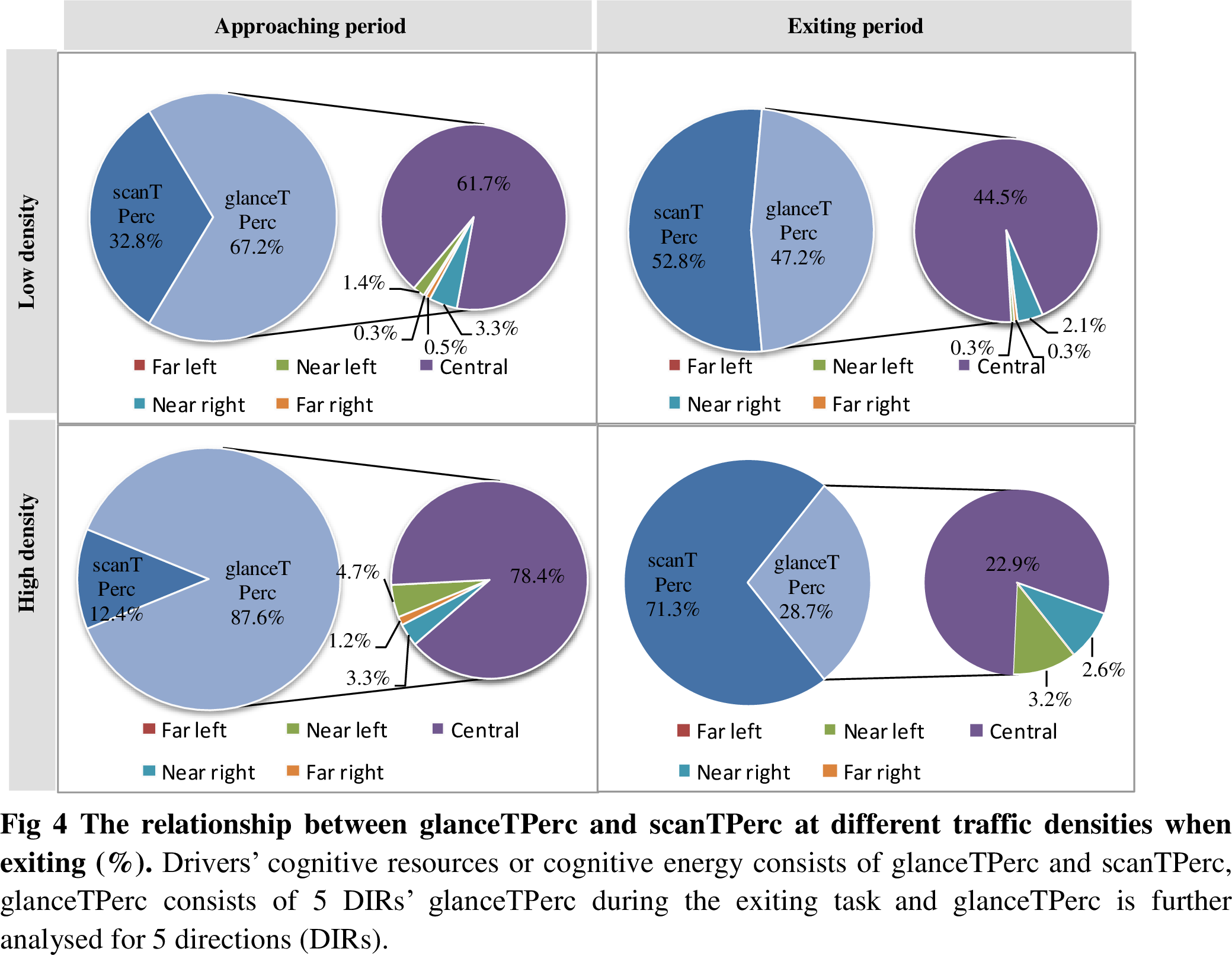

Supplement: S4 Fig — (TIF) [file pone.0162298.s004.tif]

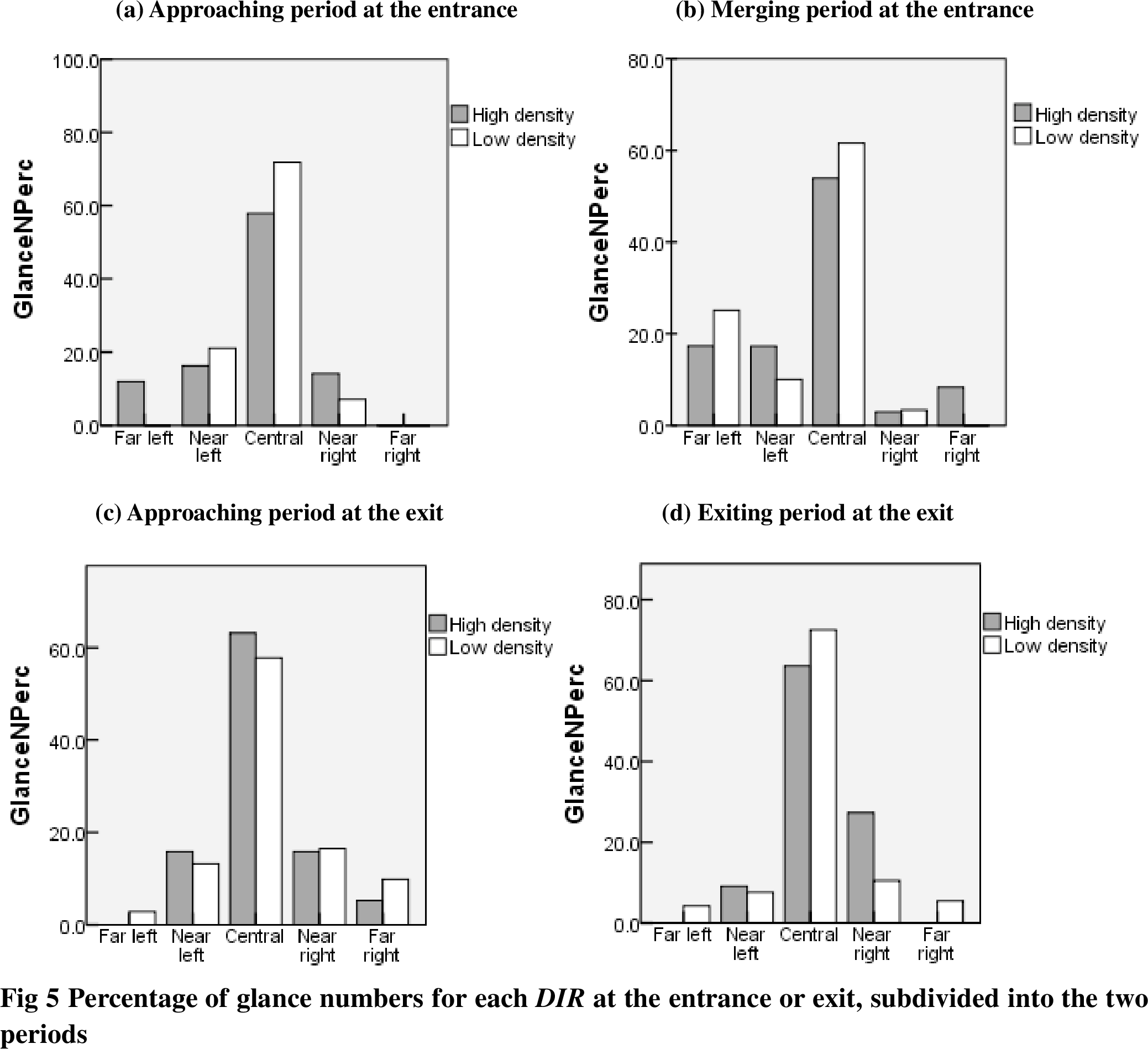

Supplement: S5 Fig — (TIF) [file pone.0162298.s005.tif]

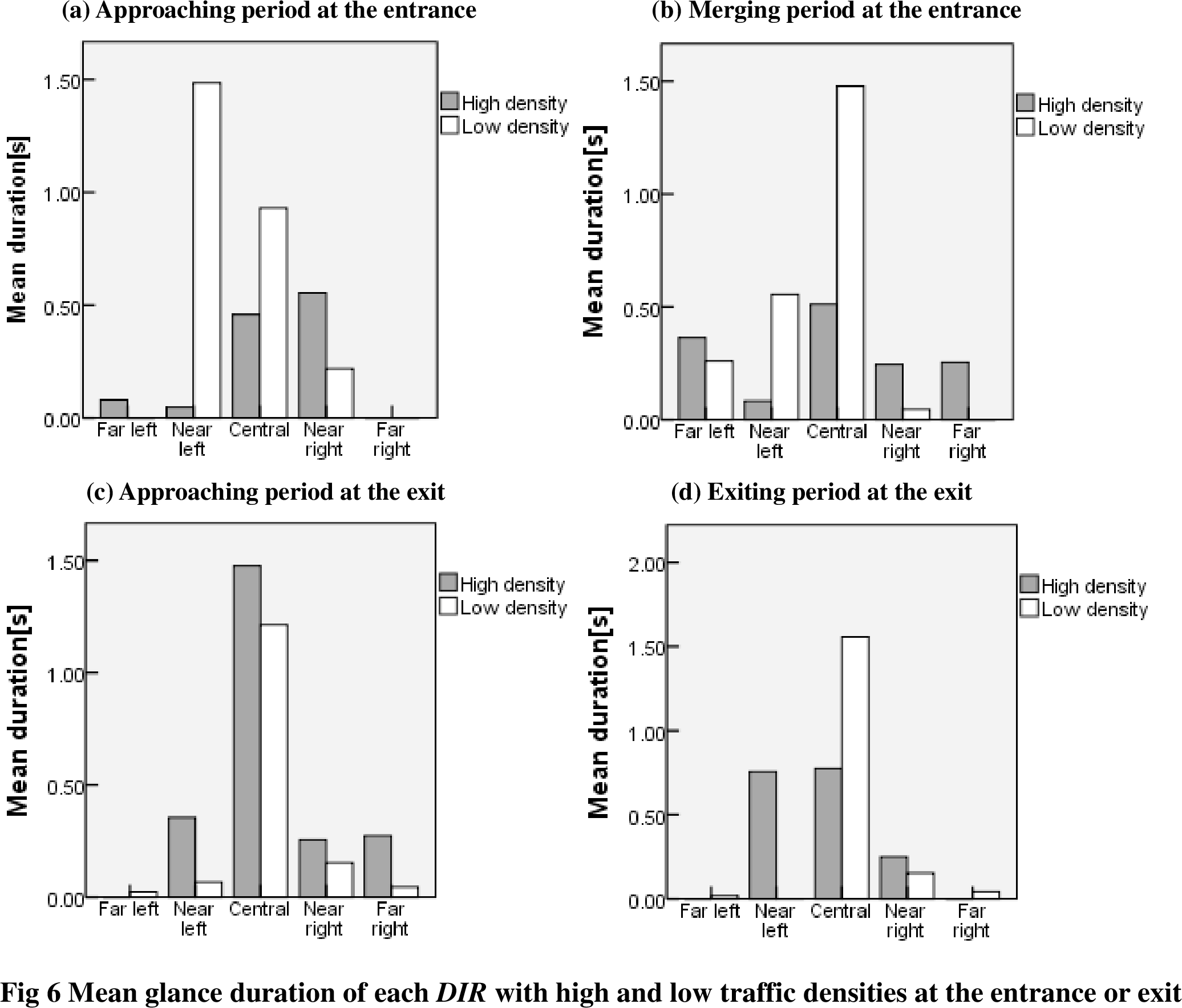

Supplement: S6 Fig — (TIF) [file pone.0162298.s006.tif]

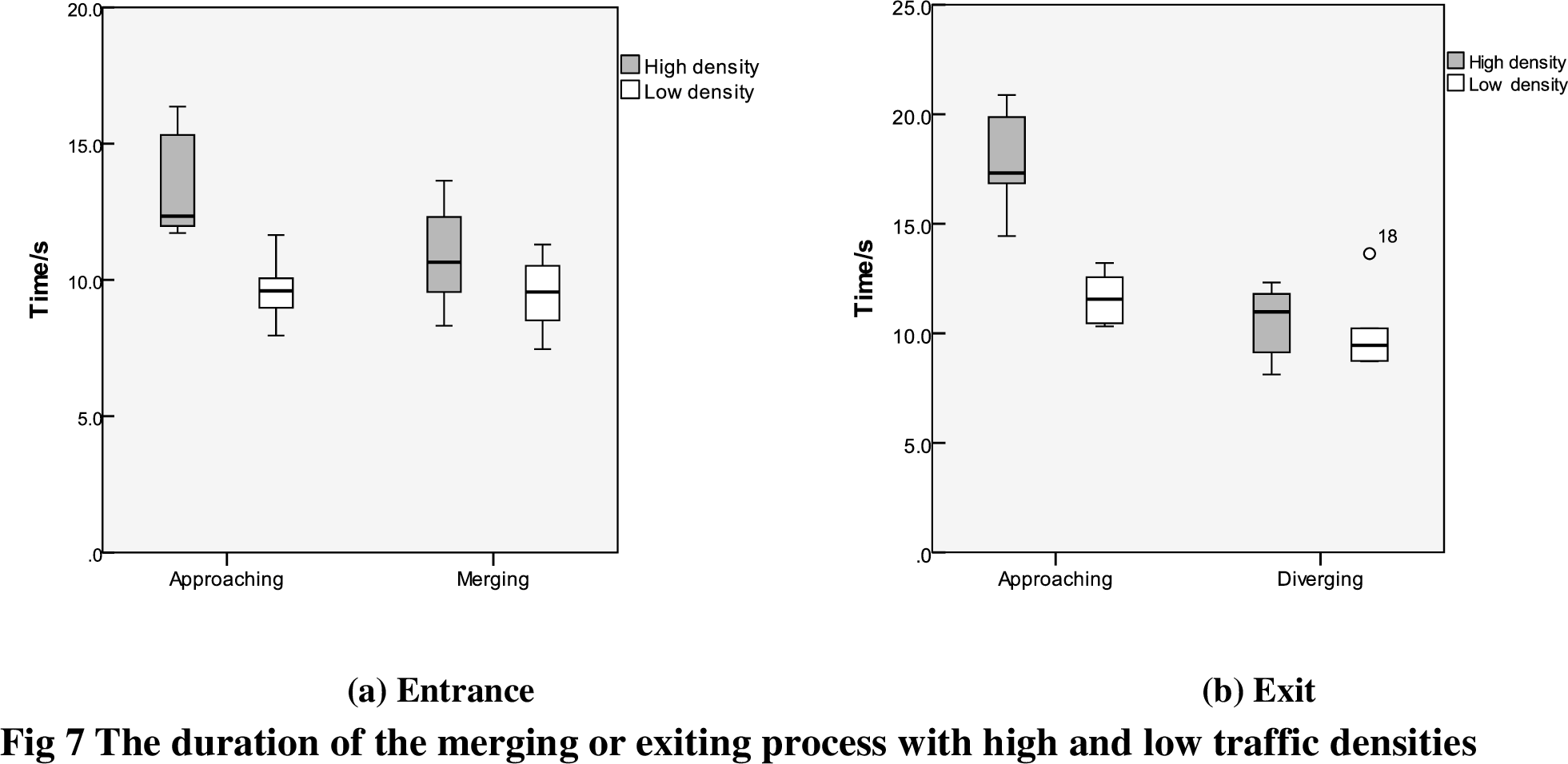

Supplement: S7 Fig — (TIF) [file pone.0162298.s007.tif]

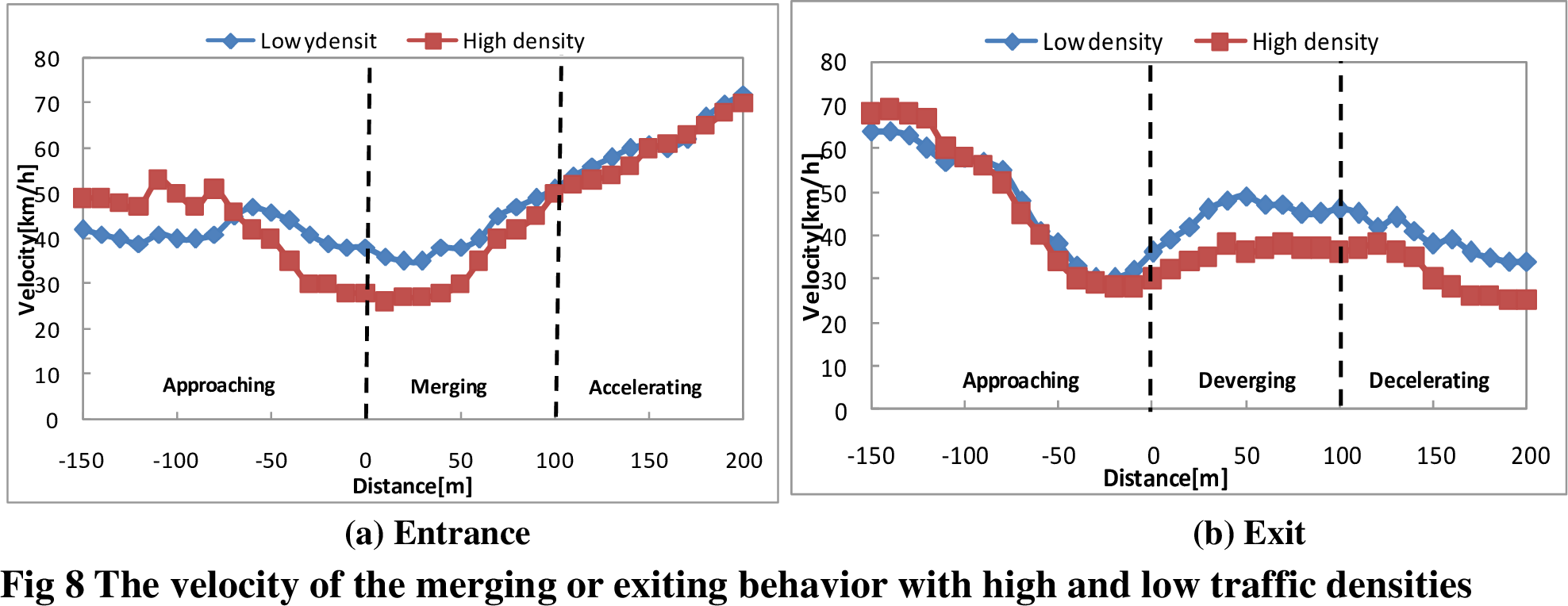

Supplement: S8 Fig — (TIF) [file pone.0162298.s008.tif]
