# Supplementary material for: Drivers’ Visual Characteristics when Merging onto or Exiting an Urban Expressway
Source: PLoS One. 2016 Sep 22;11(9):e0162298. doi: 10.1371/journal.pone.0162298 (PMC5033524; doi:10.1371/journal.pone.0162298)
Supplement: S1 Table — (DOC) [file pone.0162298.s012.doc]

**Table 1 An overview of the dependent variables analyzed in** each period

| **Period** | **Dependent variables** | |
| --- | --- | --- |
|  | **Glance data** | **Driving data** |
| ***Approaching*** | Percentages of glanceTime and scanTime (%) | Time (s) |
|  | Percentage of glance number (%) | Velocity (km/h) |
|  | Mean glance duration (s) |  |
| ***Merging or exiting*** | Percentages of glanceTime and scanTime (%) | Time (s) |
|  | Percentage of glance number (%) | Velocity (km/h) |
|  | Mean glance duration (s) |  |
| ***Accelerating or decelerating*** |  | Velocity (km/h) |
