# Supplementary material for: Drivers’ Visual Characteristics when Merging onto or Exiting an Urban Expressway
Source: PLoS One. 2016 Sep 22;11(9):e0162298. doi: 10.1371/journal.pone.0162298 (PMC5033524; doi:10.1371/journal.pone.0162298)
Supplement: S2 Table — (DOC) [file pone.0162298.s013.doc]

Table 2 Experimental regression model

| Function | Low density | High density |
| --- | --- | --- |
| Entrance | $v=\left\{\begin{matrix} \text{40 -}150<x<0 \\ 0.002x^{2}\text{ - 0.075}x\text{ + 36.65 0}\leq x<100 \\ \text{37.99e}^{0.003x}\text{ 100}\leq x<200\text{ } \end{matrix}\right.$ $R^{2}$ are 0.865, 0.954, and 0.952 respectively | $v=\left\{\begin{matrix} \text{-0.002}x^{2}\text{ - 0.497x + 20.51 -}150<x<0 \\ 0.003x^{2}\text{ - 0.060x + 26.87 0}\leq x<100 \\ \text{35.28e}^{0.003x}\text{ 100}\leq x<200\text{ } \end{matrix}\right.$ $R^{2}$ are 0.865, 0.740, and 0.691 respectively |
| Exit | $v=\left\{\begin{matrix} \text{-0.286}x\text{+ 25.78 -}150<x<0 \\ 0.003x^{2}\text{ - 0.374}x\text{ + 36.25 0}\leq x<100 \\ \text{ -25.5ln(}x\text{) + 159.2 100}\leq x<200\text{ } \end{matrix}\right.$ $R^{2}$ are 0.865, 0.740, and 0.691 respectively | $v=\left\{\begin{matrix} \text{-0.355}x\text{ + 20.4 -}150<x<0 \\ \text{-0.001}x^{2}\text{ + 0.221 }x\text{+ 30.09 0}\leq x<100 \\ \text{ -19.6ln(}x\text{) + 137.4 100}\leq x<200\text{ } \end{matrix}\right.$ $R^{2}$ are 0.865, 0.740, and 0.691 respectively |
